# One Third of Patients before Endoprosthesis Implantation Show an Oral Focus as Potential Source of Infectious Complication—The Value of Pre-Operative Dental Risk Stratification in a German Cohort

**DOI:** 10.3390/jcm11133686

**Published:** 2022-06-26

**Authors:** Gerhard Schmalz, Florentine Reuschel, Markus Bartl, Laura Schmidt, Janine Runge, Rainer Haak, Szymon Goralski, Andreas Roth, Dirk Ziebolz

**Affiliations:** 1 Department of Cariology, Endodontology and Periodontology, University of Leipzig, Liebigstraße 12, 04103 Leipzig, Germany; florentine.reuschel@web.de (F.R.); markus.bartl@medizin.uni-leipzig.de (M.B.); laura.schmidt2@medizin.uni-leipzig.de (L.S.); janine.runge@medizin.uni-leipzig.de (J.R.); rainer.haak@medizin.uni-leipzig.de (R.H.); dirk.ziebolz@medizin.uni-leipzig.de (D.Z.); 2 Department of Orthopaedics, Trauma and Plastic Surgery, University Hospital Leipzig, 04103 Leipzig, Germany; szymon.goralski@medizin.uni-leipzig.de (S.G.); andreas.roth@medizin.uni-leipzig.de (A.R.)

**Keywords:** joint replacement, oral focus, dental care, periodontitis, prevention

## Abstract

Objective: The aim of this cross-sectional cohort study was to evaluate a comprehensive dental examination and referral concept for patients prior to endoprosthesis (EP) implantation in an interdisciplinary setting. Methods: Patients, who were prepared for EP surgery in the clinic for orthopaedics, were referred to the dental clinic for a dental examination. Thereby, dental and periodontal treatment need, radiographic and temporomandibular joint findings were assessed. Based on oral and radiographic investigation, a risk classification for potential source of prosthetic infection was performed. If potential oral foci of EP infection were present (e.g., apically radiolucent teeth, severe periodontitis or additional inflammatory findings), patients were classified as at high risk for EP infection with oral origin. Those individuals were allocated to their family dentist or special clinic for dental treatment prior to EP surgery. Results: A total of 311 patients were included (mean age: 67.84 ± 10.96 years, 51% male). A dental treatment need of 33% was found, while the periodontal treatment need was 83%. Thirty-one percent of patients showed at least one apical radiolucency (a sign of chronic infection/inflammation). Furthermore, additional findings such as radiographic signs of sinusitis maxillaris were found in 24% of patients. Temporomandibular disease was probable in 17% of individuals. One-third (34%) were assigned to the high risk group for an EP infection with oral origin. Conclusion: German patients before EP have a high periodontal treatment need and show frequently (34%) a potential oral focus of infection, underlining the necessity of including dental examination and risk stratification as part of the pre-operative assessment prior to EP implantation. Therefore, an approach as applied in this study appears reasonable for those individuals.

## 1. Introduction

With more than 400,000 surgical procedures annually, knee or hip joint replacement surgery is one of the most common elective operations in hospitalized individuals in Germany [1,2]. Similarly, the implantation of endoprostheses (EP) is one of the most relevant surgical procedures in orthopaedic surgery worldwide, whereby an increase in patients can be expected based on the demographic change [3]. In most cases, long-term success of the EP therapy can be achieved; however, several complications can be observed, including aseptic loosening, dislocation and infection of the EP, causing high morbidity and necessity for difficult therapeutic interventions [4]. Accordingly, the avoidance of such complications or reducing the risk of them must be seen as an important aim in EP treatment, respectively. Thereby, patient-related outcomes such as pain reduction, function and quality of life are in the focus of care [4].

One potential cause of EP complications could be the orofacial system, including the teeth, gums, soft tissues, jaw bone and temporomandibular joint. Thus, the oral cavity as a potential source of infectious complications has been repeatedly discussed. In particular, the detection of oral disease-related microorganisms in infected EP can be seen as a hint for a relationship between oral inflammation and EP infection [5,6]. Furthermore, a high prevalence of oral diseases, especially periodontitis, in patients before and after EP implantation was observed [7,8].

It is well known that dental interventions and routine daily procedures such as flossing or tooth brushing can cause bacteraemia, which is related to the degree of periodontal inflammation [9,10]. These findings indicate that the oral cavity, especially the inflamed periodontal tissues, are a plausible and conceivable source for EP infection. Hence, the concept of an oral focus of infection, i.e., a pathologic process in the oral cavity causing no major infectious complications in healthy individuals, but having the potential to cause severe local and/or systemic complications under certain circumstances, has already been established [11,12]. However, the literature related to EP is far from consistent and clear in this context, providing limited evidence for the relevance of oral diseases or oral foci to EP infections [13,14,15]. Nevertheless, the frequent detection of a potential oral origin for EP infection on the one hand [5,6,14,16] and the high dental and periodontal treatment need of those individuals on the other hand [7] indicate that a comprehensive oral examination prior to EP implantation is required [17].

Until now, no reliable concept has been available for this issue. Although an oral examination prior to EP insertion is recommended to exclude or rehabilitate potential oral foci [18], a clear practice concept has neither been introduced nor validated, yet. However, a preoperative dental examination and respective care can be seen as the most important strategy to prevent EP infections with oral origin, in contrast to the obviously non-effective antibiotic prophylaxis for dental procedures after EP implantation [17].

Therefore, this current cohort study applied a dental examination (dental check-up) and allocation concept with a risk classification within an interdisciplinary project. This current study comprehensively evaluated the dental and periodontal treatment need alongside radiographic findings and the resulting risk groups of patients prior to EP. Hence, the need for a dental care concept should be evaluated. Accordingly, the objective of the study was to assess the potential value of including dental examination and risk stratification as part of the pre-operative assessment prior to EP implantation based on the present treatment need and occurrence of potential oral foci of EP infections. It was hypothesized that potential oral foci of EP infections are very common in the cohort, and thus, the dental care concept would be of high value.

## 2. Methods

### 2.1. Study Design

This current cohort study followed a cross-sectional design to investigate patients prior to EP insertion. For this study, the criteria as formulated in the Strengthening the Reporting of Observational Studies in Epidemiology (STROBE) statement were followed [19]. The whole study protocol was approved by the ethics committee of Leipzig University (No: 116/20-ek). All participating patients gave their written informed consent. The study was performed in full accordance with the Declaration of Helsinki.

### 2.2. Patients

Patients, who visited the Department of Orthopaedics, Trauma and Plastic Surgery, University Hospital Leipzig between April 2020 and December 2021 regarding an EP planning appointment were recruited. All individuals were informed about the study and provided their written consent for participation. Subsequently, those patients were referred for oral examination to the Department of Cariology, Endodontology and Periodontology, University of Leipzig on the same day. Inclusion criterion was the indication for a hip or knee replacement. Exclusion criteria were age < 18 years, worse general health conditions, which would not allow an oral examination, inability to undergo oral examination due to cognitive reasons (e.g., severe dementia) and acute indication of joint replacement, e.g., in context of traumata. The sample size was not calculated previously, but it was aimed to include as many patients as possible within the study period. 

### 2.3. Dental Consultation Concept

**Figure 1:**
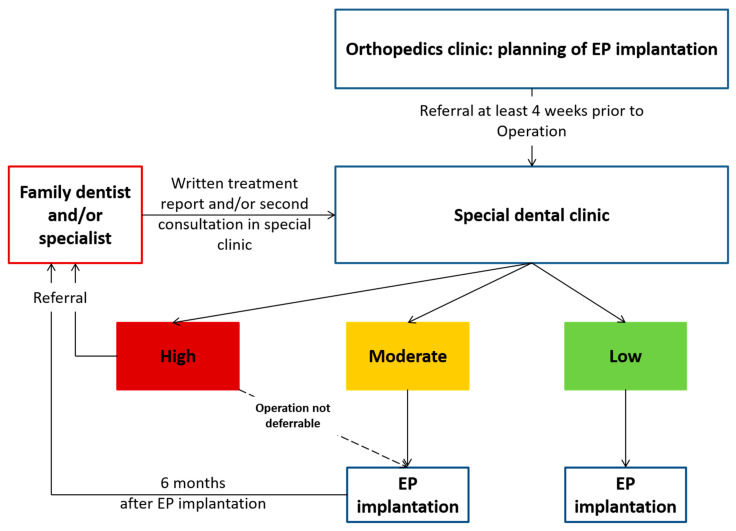
Concept of dental consultations and referral of patients before EP.

For this current study, a dental consultation concept was composed and established. This consisted of a comprehensive oral examination and, subsequently, referral of potential “at-risk” individuals for dental treatment by their family dentist or in a special dental clinic. Therefore, patients underwent a full oral examination, including dental and periodontal investigation, along with an additional radiographic diagnostic. Based on the oral findings, an individualized letter was formulated for “at-risk” patients, asking for the specific dental therapy within a defined time frame prior to surgery (not later than 2 weeks before surgery). This letter was supplemented by a response form, confirming that the respective therapy was completed. An EP insertion was only performed after confirmation of the absence of potential oral foci (criteria are listed below) or following their treatment by this signed form, respectively. The orthopaedic clinic was informed on the oral findings and risk status of the patients in writing and organized the surgery appointment accordingly. The concept is illustrated in Figure 1.

### 2.4. Oral Examination

The oral examination consisted of four parts, i.e., (I) dental, (II) periodontal and (III) radiographic examination, as well as (IV) a screening of the temporomandibular joints:

(I) Dental examination consisted of the decayed- (D-T, teeth with a carious cavitation of the tooth surface), missing- (M-T) and filled- (F-T, teeth with a dental restoration or crown) teeth index (DMF-T), which was performed visually according to WHO criteria [20].
(II) The periodontal examination consisted of the assessment of probing depth and clinical attachment loss using a periodontal probe (PCP 15/11.5B6, Hu-Friedy, Chicago, IL, USA). In combination with radiographic findings and tooth loss, the periodontitis diagnosis was determined based on the available staging and grading matrix [21]. From the periodontal probing depths, the periodontal screening index (PSI) was derived to evaluate information about periodontal condition; thereby, the presence of periodontal probing depth ≥ 3.5 < 5.5 mm (score of 3) or ≥5.5 mm (score of 4), reflected the periodontal treatment need [22,23]. Thereby, the maxilla and mandible were separated into three sextants each. If two sextants had a score of 3 or one sextant showed a score of 4, periodontal treatment need was recorded.
(III) In addition, the radiographic examination included an X-ray, regularly a panoramic radiograph. In case of difficulties to interpret the findings in the panoramic radiograph, single tooth radiographs were used, too. If patients had received radiographs in the 6 months prior to examination, these radiographs were requested from the respective dentist. All relevant structures were evaluated, including teeth, endodontium, apical and periapical region, periodontal bone, retained teeth, jawbone and sinus maxillaris.
(IV) Moreover, a screening for temporomandibular disorders (TMD) was applied, evaluating the presence of complaints and conspicuous findings of the temporomandibular joint [24].

### 2.5. Risk Classification and Further Data Collection

Based on the oral findings of the examined participants, the risk of potential EP infections with oral origin was evaluated based on the presence of treatment need and/or oral foci, respectively. Therefore, three risk classes were defined: (A) The low-risk group reflected no dental or periodontal treatment need. (B) The moderate risk group included patients with dental and/or periodontal treatment need, but no potential oral foci. (C) The high-risk group (patients “at-risk” for EP infection with oral origin) consisted of patients with a potential oral focus for EP infection, including caries, touching the pulp chamber, severe periodontal treatment need (e.g., suppuration, endo-perio-lesion), apical radiolucencies (sign of chronic infection/inflammation), (partly) retained teeth with pericoronal inflammation, inflammation in jawbone or additional inflammatory findings. These findings were reported as potential oral foci in various patient groups before and were adopted for the current study [25,26,27,28]. Those patients were referred to their family dentist/a special clinic for dental treatment, which was mandatory perquisite for EP insertion in those patients (Table 1). If the dental treatment was not possible until the time point two weeks prior to EP surgery, the EP insertion was deferred accordingly.

Within the cohort of the current study, the occurrence of early infectious complications in the first 3 months after EP insertion was recorded.

### 2.6. Statistical Analysis

Data were recorded and summarized in an Excel sheet. For analysis, mean values and standard deviations were calculated and are presented in the manuscript. Primarily, a descriptive analysis was applied. Moreover, the potential association between demographic parameters, i.e., age, gender, smoking and diabetes status with periodontal and dental treatment need as well as risk class was analysed by chi-square test. The significance level was set at *p* < 0.05.

## 3. Results

### 3.1. Patients

During the examination period, 342 patients were asked for their voluntary participation, of which 311 were included in the current study (participation rate of 91%). The mean age of the cohort was 67.84 ± 10.96 years, with about half of individuals with male gender, and about a quarter of participants were smokers (Table 2).

### 3.2. Oral Examination and Radiographic Findings

**Figure 2:**
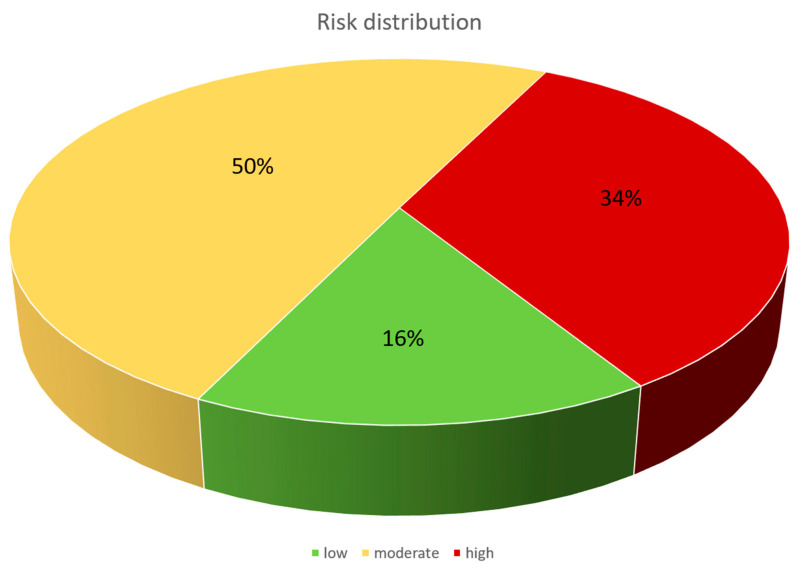
Distribution of the three risk groups, indicating a low, moderate or high risk for EP infections with oral origin.

(I) One third of individuals had at least one carious tooth, reflecting a dental treatment need of 33%. More than half of patients were denture wearers (57%), of which the minority had an insufficient denture. The vast majority of patients had a stage III or IV periodontitis (Table 3). (II) The periodontal treatment need was 83%. (III) A panoramic radiograph was performed in 291 patients. Thereby, nearly one third showed an apical radiolucency (31%). Moreover, additional findings such as radiographic signs of sinusitis maxillaris were common, as they were found in 24% of patients (Table 4). (IV) In screening of the temporomandibular joint, a TMD was probable in 17% of individuals, whereby joint noise was the most frequently detected finding (25%, Table 5). One-third (34%) of patients were classified as at high risk for an EP infection with oral origin. Half of the participants had a moderate, and 16% had a low risk (Figure 2), based on the above-mentioned classification system (see Table 1). Only four patients of the cohort developed an early infectious complication of the EP during the study period. Out of these individuals, none had a potential oral origin of the infection.

### 3.3. Associations between Demographic and Clinical Data

An increased age (age over 67 years) was associated with less periodontal treatment need (*p* = 0.02), but not with dental treatment need (*p* = 0.99). Further associations between treatment need and gender, smoking or diabetes status were not found (Table 6 and Table 7). Patients with lower mean age (<67 years), were more likely to have a high risk of oral focus-related complications (*p* < 0.01). Other associations between risk class and demographic parameters were not found (Table 8). 

## 4. Discussion

A recent large cohort study examined 9427 patients and concluded that the antibiotic prophylaxis, which is often applied to patients after EP for dental interventions, would not be effective in preventing EP infections with an oral origin. In contrast, maintaining appropriate oral health conditions was concluded to be the most suitable measure to avoid the (rarely occurring) EP infections with an oral origin [17]. Against this background, a comprehensive dental examination and referral concept was developed and evaluated in this current study, because those concept approaches are obviously needed, but still not available [18]. Within this concept, a high prevalence of dental and periodontal treatment need, along with frequent occurrence (34%) of potential oral foci for EP infections was found. Early EP infections with potential oral origin did not occur in this cohort, which received full dental examination and, in case of high risk, dental therapy (clearance of oral foci with risk of EP infection) prior to EP implantation.

A high dental and periodontal treatment need of patients prior to EP is supported by other findings in the literature. On the one hand, periodontal treatment need in the German general population is high, showing a periodontal treatment need of 75.4% in a representative population group of comparable age [29]. A Polish study confirmed a high periodontal treatment need in patients prior to EP, whereby 28.5% of individuals even had severe periodontitis [8]. Periodontal disease severity was also evaluated in the current study, showing a very high prevalence of stage III and IV periodontitis. Another Scandinavian study also confirmed a high need for dental care in patients prior to EP, requiring comprehensive dental examination and therapy prior to EP [7]. This is in line with the current study. Moreover, the current data show that radiographs appeared very reasonable in patients prior to EP, as one-third of patients showed apical radiolucency of at least one tooth, indicating a high treatment need in this respect. Furthermore, the current study showed that the high treatment need and prevalence of potential oral foci was mainly independent from demographic data. Only age was associated with increased periodontal treatment need and higher probability of high risk for infectious complications. A higher number of remaining teeth in younger individuals, and thus an increased chance to have teeth with periodontal pockets and/or apical radiolucency, might explain this. In consequence, the dental care concept would be recommendable irrespective of demographic parameters. 

The role of potential oral foci for development of EP infections is discussed controversially. In selected cases, an oral origin of bacteria colonizing the infected joint has been found [5,6,14,16]. Overall, the potential underlying mechanism seems plausible; an oral disease, such as periodontitis, leads to a transient bacteraemia [9]. In cases of severe periodontal inflammation, this is increasingly caused by the loss of integrity and thus higher permeability of the junctional epithelium [30]. In this respect, identic clones of periodontal bacteria have been detected in periodontal pockets and synovial fluid, which is especially evident for common potential pathogens such as *Fusobacterium nucleatum* [5]. However, considering the high prevalence of periodontal treatment need (>80% in the current cohort), it remains largely questionable as to why EP infections are such a rare condition if they are closely related to periodontal (and general oral) health. Based on the literature, 0.3–2% of patients develop an EP infection, of which only 3–13% are of oral origin [11,31,32]. If one estimates a prevalence of EP infections with oral origin inn all EP patients based on these numbers, this would mean a value between 0.009% (3% of 0.3% at minimum) and 0.26% (13% of 2% at maximum). This is contradictory to the enormous prevalence of oral diseases in the patients, which would cause a much higher prevalence of EP infections of oral origin if those oral conditions were highly relevant. In particular, considering the fact that most oral foci as origin of EP infections were acute exacerbations of apically inflamed teeth [31], the periodontitis-related risk of EP infection appears thus largely overestimated.

Altogether, the current concept is less a strategy to avoid EP infections and more an approach for a patient group, which is obviously dentally underserved. The high need for oral care in this patient group, especially the high prevalence of apically conspicuous teeth, was the main finding of the current study. This is in line with comparable examinations in cohorts of patients before and after EP implantation [7,8]. However, there are also works in literature that indicate that comprehensive dental examination and rehabilitation prior to EP would be an unnecessary additional expenditure, even leading to over-therapy [33]. Based on the findings of the current study, however, a dental examination and referral of at-risk patients prior to EP appears reasonable for several reasons. (I) The patients show a high oral disease burden and treatment need, which deserves a dental therapy and maintenance approach irrespective of their status as EP candidates. It has been reported that chronically ill patients, especially if their general disease burden is high, perceive a reduced awareness of oral health issues (“response shift”) [34]. Thus, those patients need to be included in a dental care concept to support their oral health behavior and physical oral status. (II) The concept fulfils the demand of dental rehabilitation and maintenance of EP patients, as pointed out in the literature [17]. A strength of the concept is the inclusion of the family dentists, while the dental clinic had more a control function to filter out patients that are at-risk for infectious complications with oral origin. (III) A potential benefit, i.e., a reduction in EP infections, might be very small, but, considering the high burden of patients with EP infections [4], even interventions with a small effect appear reasonable. This is slightly supported by the absence of oral focus-related EP infections in the current study, although this needs to be interpreted with high caution because of the low prevalence of such complications in general. It must, therefore, be mentioned that the discussion on the relevance of oral foci for EP infections would not exist if all patients had healthy/stable/inflammation-free oral conditions and were under preventive dental maintenance. (IV) Patients with EP might have underlying or co-morbidities such as rheumatic diseases, which are also related to oral, especially periodontal health [35]. Therefore, oral care for those patients with multiple risks for oral diseases appear reasonable. On the whole, an oral care approach for patients with EP, as applied in the current study, appears appropriate and needed. Taking into account that this interprofessional concept in the current study worked very well, it can be seen as a good example for future cooperation between dentistry and orthopaedic surgery.

Strengths and limitations: This cohort study evaluated a novel concept for dental care of patients prior to EP. The implication of this approach in the regular care of patients serves as a prime example to fulfil the demand, which is formulated in the literature [17]. However, several limitations need to be recognized. The sample size was high, but considering the low prevalence of EP infections, too low to draw conclusions on the effect of dental care on EP infections. Although this was not the main aim of the current study, this fact would be of practical interest. In this context, it was just a cohort study, where every participant received an “intervention”, i.e., dental care, whereby no control group was included. Moreover, co-morbidities were not assessed and considered, but could affect both the oral health and EP outcome (e.g., rheumatic diseases or medication). However, the current study showed an overall high oral disease burden, which required dental care for all of the patients prior to EP, irrespective of potential additional risk factors. This is supported by the absence of associations between demographic data and clinical findings. Therefore, the current study did not consider this issue explicitly. The concept was realized in a university setting, making the transferability to a general population questionable, and thus deserves further evaluation. Additionally, there was insufficient information on the exact dental procedures and their quality, performed by the family dentists of the participants. This should be addressed in subsequent projects. Although every dentist had to fill out a response form with a signed confirmation that the patient was free from oral foci for potential EP infection, this remained a black box in the current study. 

## 5. Conclusions

Within the limitations of the current study, patients prior to EP showed a high periodontal treatment need. Moreover, one-third of patients had a potential oral focus, underlining the high value of a dental care concept for those individuals. A dental care concept including dental examination and risk stratification as part of the pre-operative assessment prior to EP implantation, e.g., as applied in this study, appears reasonable for those patients, while its benefit for the reduction of EP infections cannot be clarified, yet.

## Figures and Tables

**Table 1:** Definition of the three risk groups for endoprosthesis (EP) infection with oral origin.

Low Risk	Moderate Risk	High Risk
No (invasive) dental or periodontal treatment need *EP implantation without any oral health considerations.*	Carious lesions with dentin exposure, with healthy pulp status Moderate periodontal treatment need, i.e., periodontal screening index (PSI) ≥ 3 in maximum of two sextants No apical radiolucency, no inflammatory findings in radiographs *EP implantation with a subsequent referral to the dentist at 6 months after EP implantation.*	Profound caries, touching the pulp chamber Severe periodontal treatment need (e.g., suppuration, endo-perio-lesion) Apical radiolucencies (partly) retained teeth with pericoronal inflammation, inflammation in jaw bone, additional inflammatory findings *EP implantation is deferred until potential dental focus is therapized. Referral to family dentist and/or specialists for demand-oriented dental therapy using standardized forms.*

**Table 2:** Characteristics of patients before endoprosthesis (EP) insertion. Values are given as mean value (mv) ± standard deviation (sd) or as percentage (%).

Parameter	Patients before EP (*n* = 311)
Age in years (mv ± sd (median))	67.84 ± 10.96 (67)
Gender male (%)	51%
Smoker (%)	24%
Diabetes mellitus (%)	26.5%

**Table 3:** Dental and periodontal treatment need and denture wearing of patients before endoprosthesis (EP). The periodontitis diagnosis is given as percentage of dentate individuals.

Parameter		Patients before EP (*n* = 311)
DMF-T		20.63 ± 6.32
D-T		0.74 ± 1.49
M-T		9.83 ± 8.49
F-T		10.13 ± 6.03
Periodontal treatment need (%)		83%
Periodontitis stage (%)	Stage I	0%
	Stage II	1%
	Stage III	41%
	Stage IV	58%
Grade (%)	A	0%
	B	80%
	C	20%
Dental treatment need (%)		33%
Denture wearing (%)		57%
Insufficient denture (% of denture wearers)		16%

DMF-T: Decayed—(D-T), Missing—(M-T) and Filled-Teeth—(F-T) index.

**Table 4:** Radiological findings of patients before endoprosthesis (EP).

Finding	Patients before EP (*n* = 291)
Apical radiolucency	31%
Retained wisdom teeth without signs of inflammation	13%
Inflammatory processes of the bone	7%
Periodontal bone loss	85%
Retained wisdom teeth without signs of inflammation	5%
Additional findings	24%

**Table 5:** Findings of screening for temporomandibular dysfunction (TMD) of patients before endoprosthesis (EP).

Parameter	Patients before EP (*n* = 311)
Asymmetric mouth opening	16%
Joint noise	25%
Painful palpation of muscles	2%
Limited mouth opening	2%
Occlusal noise	4%
Traumatic eccentricity	15%
TMD probable	17%

**Table 6:** Associations between demographic data and periodontal treatment need.

		Periodontal Treatment Need		
		Yes	No	*p*-Value
Age	<67 years	51.7%	26.7%	0.02
	≥67 years	48.3%	73.3%	
Gender	Male	47.7%	56.7%	0.43
	Female	52.3%	43.3%	
Smoking	Yes	25.7%	17.9%	0.48
	No	73.3%	82.1%	
Diabetes	No	73.3%	76.7%	0.27
	Yes, HbA1c < 7%	14%	20%	
	Yes, HbA1c ≥ 7%	12.7%	3.3%	

**Table 7:** Associations between demographic data and dental treatment need.

		Dental Treatment Need		
		Yes	No	*p*-Value
Age	<67 years	53.4%	47.6%	0.99
	≥67 years	46.6%	52.4%	
Gender	Male	60.3%	46.8%	0.11
	Female	39.7%	53.2%	
Smoking	Yes	29.6%	21.8%	0.34
	No	70.4%	78.2%	
Diabetes	No	77.2%	65.5%	0.24
	Yes, HbA1c < 7%	13%	20.7%	
	Yes, HbA1c ≥ 7%	9.8%	13.8%	

**Table 8:** Associations between demographic data and risk stratification.

		Risk Class			
		Low	Moderate	High	*p*-Value
Age	<67 years	75.6%	50.7%	56.5%	<0.01
	≥67 years	24.4%	49.3%	43.5%	
Gender	Male	36.6%	56.5%	55.1%	0.10
	Female	63.4%	43.5%	44.9%	
Smoking	Yes	17.9%	22.7%	27.7%	0.52
	No	82.1%	77.3%	72.3%	
Diabetes	No	4.9%	7.4%	18.8%	0.09
	Yes, HbA1c < 7%	22%	13.2%	13.0%	
	Yes, HbA1c ≥ 7%	73.1%	79.4%	68.1%	

## Data Availability

The data presented in this study are available on request from the corresponding author. The data are not publicly available due to ethical concerns.
